# Etymologia: *Shewanella haliotis*

**DOI:** 10.3201/eid1906.ET1906

**Published:** 2013-06

***Shewanella haliotis* [shooʺə-nelʹə hāʺlĭ-oʹtĭs]**

From the Greek *halios* (marine) and *ōtos* (ear), abalones, genus *Haliotis*, were first mentioned ≈2,500 years ago by Aristotle, who wrote of “the wild limpet (called by some the ‘sea ear’).” In D’Arcy Thompson’s translation of Aristotle, he notes that “wild limpet” is “commonly attributed to *Fissurella graecea* ... and conceals a forgotten name for *Haliotis*.” The “sea ear” was familiar to the Greeks and was named *otia* (little ear) by Pliny.

*Shewanella haliotis*, a species of rod-shaped, gram-negative, facultatively anaerobic bacteria, was first isolated from the gut microflora of abalones collected from the ocean near Yeosu, South Korea, by Kim et al. in 2007. The genus *Shewanella* had been previously named in 1985 by MacDonell and Colwell in honor of Scottish microbiologist James M. Shewan, for his work in fisheries microbiology.
